# Mitarbeiterbefragung nach Einführung der elektronischen Patientenakte FIDUS an der Universitätsaugenklinik des Saarlandes

**DOI:** 10.1007/s00347-021-01514-1

**Published:** 2021-10-27

**Authors:** Amine Maamri, Fabian N. Fries, Corinna Spira-Eppig, Timo Eppig, Berthold Seitz

**Affiliations:** Klinik für Augenheilkunde, Universitätsklinikum des Saarlandes (UKS), Kirrberger Straße 100, Gebäude 22, 66421 Homburg/Saar, Deutschland

**Keywords:** Augenheilkunde, Optimierung, Digitalisierung, Kooperationsportal, Kontinuierlicher Verbesserungsprozess, Ophthalmology, Optimization, Digitalization, Continuous improvement process

## Abstract

**Ziel:**

Ziel der Studie war es, die Zufriedenheit der Mitarbeiterinnen und Mitarbeiter der Universitätsaugenklinik des Saarlandes mit der elektronischen Patientenakte FIDUS zwischen Dezember 2016 und September 2020 zu vergleichen, nachdem sie im Januar 2016 eingeführt worden war.

**Methoden:**

Um diese Studie durchzuführen, hat das FIDUS-Team der Universitätsaugenklinik des Saarlandes einen Fragebogen erstellt. Dieser wurde im Dezember 2016 und im September 2020 an alle Beteiligten verteilt: ärztliches Personal, Pflege- und Verwaltungspersonal. Der Bogen enthielt 19 Fragen, die auf einer Skala von 0 für („stimme nicht zu“) bis 10 („stimme vollständig zu“) zu beantworten waren.

**Ergebnisse:**

Die Anzahl der Teilnehmenden hatte von 60 (44 %) auf 89 (64 %) zwischen 2016 und 2020 deutlich zugenommen. Davon waren 23 (25,8 %) dem ärztlichen und 27 (30,3 %) dem pflegerischen Personal zuzuordnen, 12 (13,4 %) waren Verwaltungsbereich, 16 (17,9 %) in einem sonstigen Arbeitsverhältnis, und 11 (12,3 %) machten keine Angabe. Im Jahr 2020 haben 75,6 % der Befragten auf der Skala mit „10“ zugestimmt, dass die Einführung der elektronischen Patientenakte der richtige Schritt war, im Vergleich zu 36,7 % im Jahr 2016 (*p* < 0,001). Die Höchstbewertung, dass „die Akteneinträge mit dem Computer schneller zu schreiben sind als handschriftlich“, lag bei 38 % im Jahr 2020 vs. 25 % im Jahr 2016 (*p* < 0,001). Im Jahr 2020 befürworteten 61,6 % der Befragten vollständig die Einführung der papierlosen Patientenaufklärung als nächsten Schritt.

**Schlussfolgerung:**

Die Zufriedenheit mit der elektronischen Patientenakte FIDUS hat sich im Jahr 2020 im Vergleich zu 2016 signifikant verbessert. Das gilt insbesondere für die bessere Übersichtlichkeit der Akte und die schnelleren Arbeitsabläufe. Allerdings scheint noch Optimierungsbedarf im Detail zu bestehen.

## Abstract (en)

**Aim:**

This study compared Saarland University Eye Hospital employees’ satisfaction with the FIDUS electronic patient record between December 2016 and September 2020, after its introduction in January 2016.

**Methods:**

For the retrospective study, the FIDUS team at the Saarland University Eye Hospital created a questionnaire which was distributed to all participants (physicians, nursing staff, and administrative staff) in December 2016 and September 2020. The questionnaire contained 19 questions, each scored from 0 to 10 (0: disagree; 5: partly agree; 10: completely agree).

**Results:**

The number of participants increased significantly from 60/136 (47%) to 89/140 (64%) between 2016 and 2020. Of these participants, 23 (25.8%) were physicians, 27 (30.3%) were nurses, 12 (13.4%) were in administration, 16 (17.9%) were in other employment, and 11 (12.3%) did not disclose their employment. In 2020, 75.6% of employees agreed with a score of 10 that implementation of electronic medical records was the right step, compared to only 36.7% in 2016 (*p* < 0.001). The highest rating for “record entries are faster to write with a computer than handwritten” was obtained in 38% in 2020 vs. 25% in 2016 (*p* < 0.001).

**Conclusion:**

Satisfaction with the FIDUS electronic patient record has improved significantly in 2020 compared to 2016, particularly concerning file clarity and faster workflows. The proportion of respondents who see their initial expectations of electronic patient files as 100% fulfilled has tripled. However, there is still a need for optimization of details.

## Hintergrund

Heutzutage ist die Digitalisierung in jeder Branche unabdingbar. Auch die Augenheilkunde hat in den vergangenen Jahren rasante Entwicklungen zu verzeichnen [2, 12, 14]. Neben der mittlerweile überwiegend elektronischen Dokumentation und Organisation steht eine Vielzahl von bildgebenden Diagnostikverfahren zur Verfügung [3, 10].

Mit dem übergeordneten Ziel, die Patientenversorgung und die Kommunikation mit niedergelassenen Kolleginnen und Kollegen sowie anderen Kliniken zu verbessern, wurde nach mehr als 60 Jahren handschriftlich geführten Papierakten an der Universitätsaugenklinik des Saarlandes (UKS) am 01. Januar 2016 eine elektronische Patientenakte (EPA) auf Basis von FIDUS (Arztservice Wente GmbH, Darmstadt) eingeführt [12, 13]. Im September 2017 folgte das Kooperationsportal „UKS.AUGEN.NETZ“ auf Basis des FIDUSweb [5]. Die primären Ziele der Klinik waren dabei die Optimierung der Terminvereinbarung und die schnellere Versendung der Befundbriefe. Für die an das UKS.AUGEN.NETZ angeschlossenen Ärzte sind im Terminplaner in jeder Spezialsprechstunde Termine vergeben. Über FIDUS können Briefe geschrieben, elektronisch vidiert und anschließend direkt an den kooperierenden Kollegen über das UKS.AUGEN.NETZ versendet werden [5].

Durch komfortable Unterstützung der Beteiligten soll der Aufwand für die Kommunikationsprozesse auf ein Minimum reduziert werden, um Zeit für die Behandlung der Patienten zu schaffen.

Obwohl für dieses große Projekt von Anfang an breiter Konsens in der Klinik bestand, stellte die Einführung einer EPA und Abschaffung der papiergebundenen Dokumentation vor allem im ersten halben Jahr eine große Belastung für Teile des Personals, insbesondere für die Assistenzärztinnen und -ärzte, dar.

Die Studie von De Dreu et al. stellt eine Metaanalyse unter anderem über die Zusammenhänge zwischen Aufgabenkonflikt, Teamleistung und Zufriedenheit der Teammitglieder vor. Sowohl ein geringerer Aufgabenkonflikt als auch eine höhere Zufriedenheit der Teammitglieder soll sich positiv auf die Teamleistung auswirken. Die Zustimmung zu den in unserer Studie abgefragten Items kann daher im weiteren Sinne als Maß für den Aufgabenkonflikt und die Zufriedenheit der Teammitglieder mit den angebotenen Lösungen verstanden werden [6].

## Ziel der Studie

Ziel der Studie war es, die Zufriedenheit der Mitarbeiterinnen und Mitarbeiter der Universitätsaugenklinik des Saarlandes mit der elektronischen Patientenakte FIDUS zwischen Dezember 2016 und September 2020 zu vergleichen.

## Methoden

Mehr als 60 Jahre lang führte die Klinik für Augenheilkunde am Universitätsklinikum des Saarlandes (UKS) handschriftliche Papierakten. Wachsende Dokumentationspflichten sowie sprunghaft zunehmende elektronische Diagnostikdaten führten zu überfüllten Archiven und schwer auffindbaren Akten. Dies waren wesentliche Gründe zur Einführung der elektronischen Patientenakte (EPA).

Zwischen 2016 und 2020 wurden alle Mitarbeiterinnen und Mitarbeiter der Universitätsaugenklinik des Saarlandes stets motiviert, Verbesserungsvorschläge, Kritiken und Meinungen zur Vereinfachung von Arbeitsabläufen mit FIDUS einzureichen. Daher wurde an der Augenklinik ein FIDUS-Team von ärztlichen und nichtärztlichen Mitarbeiterinnen und Mitarbeitern gegründet. Dieses Team kümmerte sich fortwährend um die Anpassung sowie Weiterentwicklung von FIDUS und meldet zeitnah die kumulierten Fehlermeldungen und Veränderungsvorschläge an den Softwarehersteller. Durch die beständige Zusammenarbeit der Klinik für Augenheilkunde der Universitätsklinik des Saarlandes und dem Hersteller der Software kann eine kontinuierliche Optimierung von FIDUS auch bei laufendem Klinikbetrieb erfolgen und die Zusammenarbeit zwischen Klinik und niedergelassenen Praxen weiter erleichtert werden. Die Lösungsvorschläge werden vom Softwarehersteller umgesetzt und in einer Softwaredemonstration den Mitarbeitern vorgestellt. In einer gemeinsamen Diskussion werden diese anschließend gezielt an die Anforderungen angepasst [12]. Um die Zufriedenheit mit der elektronischen Patientenakte FIDUS objektiv und konkret auszuwerten, hatte das FIDUS-Team hierfür einen Fragebogen vorbereitet.

Dieser Fragebogen wurde nach Genehmigung durch Personalrat und Personaldezernat im September 2020 allen beteiligten Mitarbeiterinnen und Mitarbeitern persönlich ausgehändigt: ärztliches Personal, Schwestern, Pfleger und Mitarbeiter im Bereich der Verwaltung (Anmeldung/Archiv/Sekretariat). Der Fragebogen wurde an jeden Mitarbeiter persönlich adressiert, und die Abgabe erfolgte anonym durch Einwurf in das Postfach des Sekretariats.

Der Fragebogen wurde nach den Bedürfnissen der Mitarbeiter erstellt und konzentriert sich auf die nach der Installation der Software festgestellten Mängel. Die Fragen wurden auch durch Vorschläge der Mitarbeiter, die wir während des Prozesses erhielten, inspiriert. Wir haben unseren Fragebogen nicht von einem anderen Fragebogen in der Literatur kopiert. Es war unser eigener Fragebogen, der an die Bedürfnisse unserer Klinik angepasst wurde. Die Fragen basierten auf drei Hauptthemen: Erwartung, Arbeitsabläufe, Kommunikation und Transparenz.

Durch diesen Fragebogen hatten alle Mitarbeiterinnen und Mitarbeiter die Möglichkeit, ihr Feedback zur elektronischen Patientenakte zu geben. Ihr Feedback ist für den kontinuierlichen Verbesserungsprozess von *FIDUS* essenziell. Die Beantwortung aller Fragen war freiwillig und wurde anonym ausgewertet. Der Bogen enthielt 19 Fragen, also eine zusätzliche Frage im Vergleich zur ersten Umfrage von Dezember 2016. Jede Aussage konnte auf einer Skala von 0 bis 10 bewertet werden, wobei 0 eine absolute Ablehnung („stimme gar nicht zu“) und 10 eine vollständige Zustimmung („stimme vollständig zu“) bedeutet und 5 eine partielle Zustimmung bzw. Ablehnung („stimme teils zu“) darstellt (Tab. 1).

**Table 1:** 

Frage 19: Die Einführung der papierlosen Patientenaufklärung ist ein wichtiger nächster Schritt	2016	2020
0 (= stimme gar nicht zu)	–	1
1–4	–	1
5 (= stimme teils zu)	–	4
6–9	–	22
10 (= stimme vollständig zu)	–	45
Insgesamt	–	73

Die gesammelten Daten wurden mit Microsoft Excel (Microsoft Office, Redmond, WA, USA, 2016) analysiert und im Vergleich dargestellt. Die Mitarbeiter hatten von September bis Oktober 2020 Zeit, ihren ausgefüllten Fragebogen sowie ihre eigenen Anmerkungen einzureichen. Pro Mitarbeiter wurde ein Fragebogen ausgehändigt, die Auswertung erfolgte anonym. Um einen signifikanten Unterschied zwischen der ersten und zweiten Befragung festzustellen, wurde der Pearson‑χ^2^-Test auf Unabhängigkeit verwendet. Bei einem *p*-Wert von < 0,05 wurde ein signifikanter Unterschied zwischen den Befragungen angenommen.

## Ergebnisse

**Figure 1:**
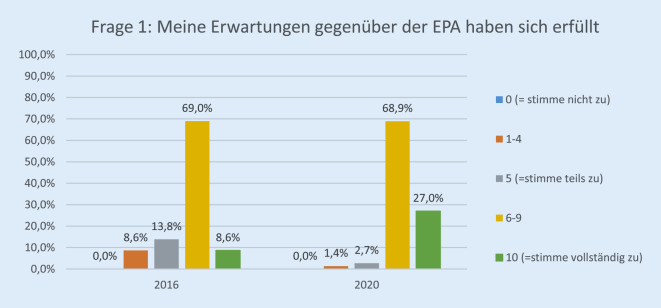

**Figure 2:**
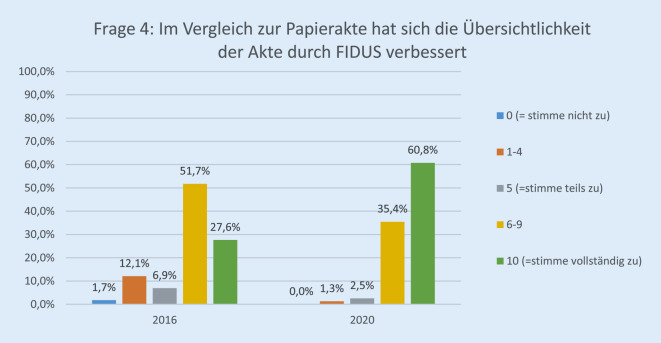

**Figure 3:**
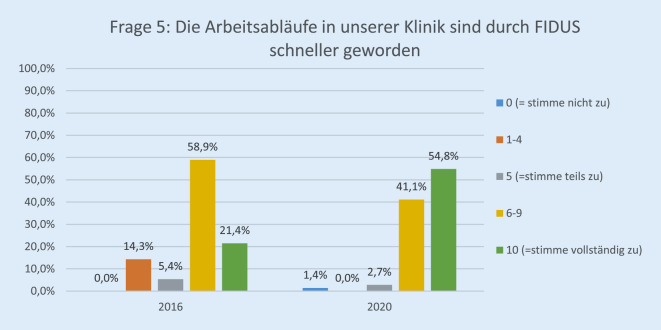

**Figure 4:**
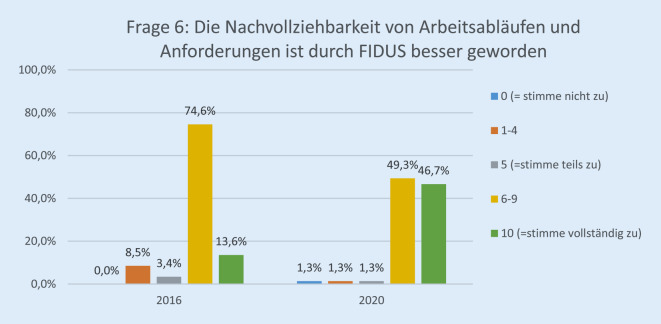

**Figure 5:**
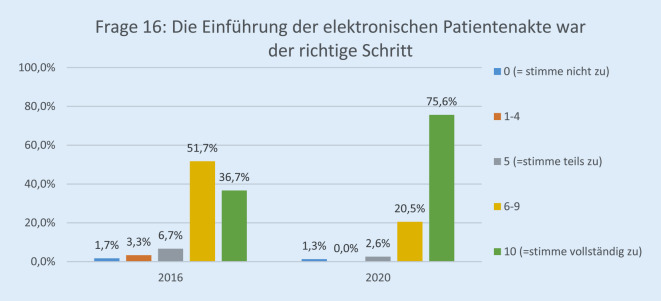

**Figure 6:**
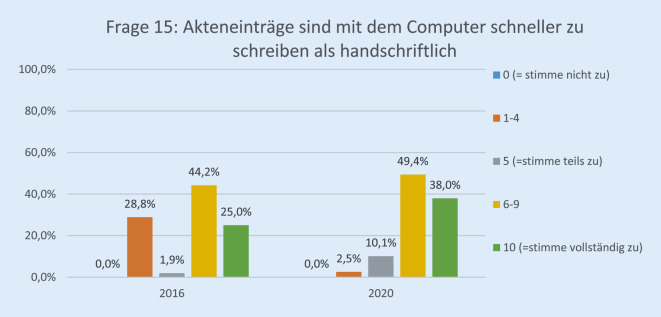

**Figure 7:**
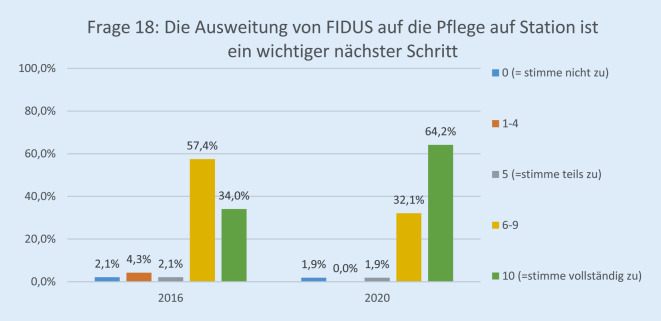

Die Rücklaufquote der Fragebögen stieg von 60 von 136 (44 %) im Jahr 2016 auf 89 von 140 (64 %) im Jahr 2020. Davon waren 25,8 % (23) ärztliches Personal, 30,3 % (27) Pflegepersonal, 13,4 % (12) Verwaltungspersonal, 17,9 % (16) in einem sonstigen Arbeitsverhältnis und 12,3 % (11) ohne Angabe. Bezüglich der Erwartung der Mitarbeiterinnen und Mitarbeiter gegenüber der EPA lag die Höchstbewertung („10“) bei 27,0 % im Jahr 2020 vs. 8,6 % im Jahr 2016 (*p* < 0,001; Abb. 1). Im Vergleich zur Papierakte hat sich die Übersichtlichkeit der Akte durch FIDUS laut 60,8 % der Mitarbeiterinnen und Mitarbeiter im Jahr 2020 im Vergleich zu 27,6 % (2016) signifikant verbessert (*p* < 0,001; Abb. 2). Hinsichtlich der schnelleren Arbeitsabläufe in unserer Klinik hat die Höchstbewertung von 21,4 % auf 54,8 % (*p* < 0,001) zugenommen (Abb. 3). Zu Frage Nummer 6 finden ca. 47 % der Mitarbeiter im Jahr 2020 im Vergleich zu ca. 14 % im Jahr 2016, dass die Nachvollziehbarkeit von Arbeitsabläufen und Anforderungen dank FIDUS besser geworden sei (*p* = 0,0005; Abb. 4). Hinsichtlich der Transparenz der medizinischen Entscheidungen in unserer Klinik durch FIDUS lag die Höchstbewertung bei 42,3 % im Jahr 2020 und bei 22,4 % im Jahr 2016 (*p* = 0,033). Laut 40,3 % der Befragten im Jahr 2020 vs. 30,5 % im Jahr 2016 (*p* = 0,003) habe FIDUS zu Zeitersparnissen im direkten Arbeitsumfeld geführt. Im Jahr 2020 haben 40,7 % der Mitarbeiter vs. 25,0 % im Jahr 2016 (*p* = 0,033) mit „10“ auf der Skala zugestimmt, dass FIDUS die tägliche Arbeit vereinfacht. Im Jahr 2020 haben 75,6 % der Mitarbeiter mit „10“ auf der Skala zugestimmt, dass die Einführung der elektronischen Patientenakte der richtige Schritt war, im Vergleich zu 36,7 % im Jahr 2016 (*p* < 0,001, Abb. 5). Im Jahr 2020 denken 49,9 % der Befragten vs. 22,0 % im Jahr 2016 (*p* = 0,02271) mit einer Höchstbewertung, dass sich die Arbeit mit der EPA noch weiter verbessern wird. Die Höchstbewertung, dass „die Akteneinträge mit dem Computer schneller zu schreiben sind als handschriftlich“ lag bei 38 % im Jahr 2020 vs. 25 % im Jahr 2016 (*p* < 0,001; Abb. 6). Außerdem ist mehr als die Hälfte der Mitarbeiterinnen und Mitarbeiter im Jahr 2020 völlig überzeugt, dass die Ausweitung von FIDUS auf die stationäre, pflegerische Dokumentation ein wichtiger nächster Schritt sein wird (*p* = 0,033; Abb. 7); im Jahr 2016 war nur ein Viertel davon überzeugt. Bei 6 Fragen gab es keinen signifikanten Unterschied bei den Antworten zwischen 2016 und 2020. Diese Fragen befassen sich mit dem reibungslosen Ablauf von FIDUS (*p* = 0,321), Umgang mit Problemen bei der Einführung (*p* = 0,576), der Verbesserung der Kommunikation in der Klinik durch FIDUS (*p* = 0,220), der Erleichterung der Abrechnung (*p* = 0,452), dem Einscannen der Altakten durch DMI (*p* = 0,516) und der Tagesakten durch ZAM (*p* = 0,422).

Die letzte Frage „Die Einführung der papierlosen Patientenaufklärung ist ein wichtiger nächster Schritt“ wurde 2020 hinzugefügt. Daher gab es keinen Vergleich mit 2016. Hier haben mehr als 60 % der befragten Mitarbeiter mit „10“ zugestimmt und mehr als 30 % mit „6–9“ (Tab. 2).

**Table 2:** 

*Erwartungen*
Meine Erwartungen gegenüber der EPA haben sich erfüllt
Die FIDUS-Einführung verlief in der Anfangsphase weitestgehend reibungslos
Den im Zuge der Einführung aufgetretenen Problemen wurde konstruktiv begegnet
Im Vergleich zur Papierakte hat sich die Übersichtlichkeit der Akte durch FIDUS verbessert
Die Einführung der elektronischen Patientenakte war der richtige Schritt
Ich denke, dass sich die Arbeit mit der EPA noch weiter verbessern wird
Die Ausweitung von FIDUS auf die Pflege auf Station ist ein wichtiger nächster Schritt
Die Einführung der papierlosen Patientenaufklärung ist ein wichtiger nächster Schritt
*Arbeitsabläufe*
Die Arbeitsabläufe in unserer Klinik sind durch FIDUS schneller geworden
Die Nachvollziehbarkeit von Arbeitsabläufen und Anordnungen ist durch FIDUS besser geworden
FIDUS hat zu Zeitersparnissen in meinem direkten Arbeitsumfeld geführt
FIDUS vereinfacht meine tägliche Arbeit
Die Abrechnung wurde durch FIDUS erleichtert
Das Einscannen der alten Akten durch einen externen Archivierungsdienstleister (DMI GmbH & Co. KG, Münster) läuft gut
Das Einscannen der Tageskarte beim zentralen Aktenmanagement (ZAM) läuft gut
Briefe werden durch FIDUS schneller fertiggestellt (verfassen, vidieren, versenden)
Akteneinträge sind mit dem Computer schneller zu schreiben als handschriftlich
*Kommunikation und Transparenz*
FIDUS erhöht die Transparenz der medizinischen Entscheidungen, die in unserer Klinik getroffen werden
FIDUS hat die Kommunikation in unserer Klinik verbessert

## Diskussion

Elektronische Patientenakten wurden nicht nur in Deutschland genutzt. Lim und Shahid berichten, dass in Großbritannien bereits mehr als 45 % der augenärztlichen Versorgungszentren über eine elektronische Patientenakte verfügen [9].

Die Ergebnisse des Fragebogens von Oktober 2020 zeigen sowohl deutliche Fortschritte und Vorteile seit der Einführung von FIDUS sowie eine kritische Selbstreflexion im Vergleich zur Situation im Dezember 2016 auf. Beim Vergleich von Papierakte und EPA hat sich die Häufigkeit der Höchstbewertung innerhalb von 4 Jahren verdoppelt. Hier zeigte sich klar, dass die Organisation der Patientenakten in FIDUS als deutlich verbessert wahrgenommen wird. Das Risiko einer Falschmedikation aufgrund unleserlicher Schrift ist nur noch theoretischer Natur.

Ein anderer Vorteil von FIDUS sind die optimierten Arbeitsabläufe in unserer Klinik. Ein gutes Beispiel dafür ist unser separates IVOM-Zentrum, wo Patientinnen und Patienten nach der intravitrealen operativen Medikamenteneingabe (IVOM) dank der EPA sofort ihren computergestützt generierten Arztbrief mit allen Informationen für die (meist externe) Nachkontrolle mitnehmen können [1].

Daher hat sich die Häufigkeit der Höchstbewertung (10) bei der fünften und siebten Frage verdoppelt. Dies könnte durch verschiedene Faktoren erklärt werden. Zum einen stehen alle Unterlagen von Patienten im Computer ortsungebunden zur Verfügung. Dies erlaubt einen schnellen Zugang, ohne im Archiv die alten Papierakten suchen zu müssen, die oft schwer auffindbar waren. Zum anderen haben Kliniken und niedergelassene Augenärzte, die FIDUS nutzen, die Möglichkeit, über eine direkte Export- und Importfunktion aus der elektronischen Patientenakte den Befundtransfer automatisch zu generieren. Bei der Nutzung anderer Arztinformationssysteme können in den Praxen Befunde als Portable Document Format (PDF) importiert oder bei einer Papierakte als Ausdruck generiert werden. Die Dateneingabe und -pflege kann dann jeweils online über die FIDUSweb-Oberfläche erfolgen. Auch in diesen Fällen kann das Anlegen der Patienten mittels Anbindung eines Kartenlesegeräts erfolgen. Die Bildgebung kann ggf. auch über Schnittstellen einzelner Diagnostikgerätehersteller mit eigener „Viewer-Software“ übermittelt werden. Hier können z. B. optische Kohärenztomographien (OCT) oder Fluoreszenzangiographien (FAG) im proprietären Herstellerformat oder als Video übertragen werden. Dies funktioniert genauso mit Standardformaten wie PDF oder JPG [5]. In der Umfrage schlägt sich das in den Antworten zu der Frage „FIDUS vereinfacht meine tägliche Arbeit“ nieder. Hier haben 40 % der Teilnehmerinnen und Teilnehmer vollkommen zugestimmt.

Im Pflegebereich sind die Akten sowohl handschriftlich als auch elektronisch. Die Medikamentenkurve, die Krankenkassenunterlagen sind in den schriftlichen Akten. Allerdings sind die schriftlichen Akten gut kontrolliert und erlauben einen niederschwelligen Einblick. Zurzeit sind wir in unserer Klinik dabei, MEONA-Akten (MEONA GmbH, Freiburg im Breisgau, Deutschland) zu installieren. Sie sind die zukünftigen elektronischen Medikamentenakten in unserer Augenklinik, aber auch an der Universitätsklinik des Saarlandes. Auch die digitale Patientenaufklärung wird zusammen mit der digitalen Datenschutzerklärung im Jahr 2021 von einem anderen Anbieter im UKS eingeführt.

Ein anderer Vorteil von FIDUS ist deutlich im Bereich der Lehre und der Forschung zu bemerken. Wir haben seit Jahren in unserer Augenklinik in Homburg daran gearbeitet, die Lehre für die Studierenden (inklusive im Praktischen Jahr, PJ) und die Weiterbildung für die Assistenzärztinnen und -ärzte zu standardisieren und zu optimieren [7, 8, 11]. Der Augenblock stellt eine nachhaltige und zukunftsorientierte Verbesserung der Lehre im Fach Augenheilkunde an der medizinischen Fakultät der Universität des Saarlandes dar. Die Einführung der EPA war nicht nur im Kontext der verbesserten Patientenversorgung, sondern auch im Hinblick auf die optimierte Assistenzarztweiterbildung ein wesentlicher Fortschritt. Die Weiterbildung an einer großen Universitätsaugenklinik besteht nicht nur aus klinischer Arbeit am Patienten sowie Nacht- und Wochenenddiensten. Auch organisatorische Aufgaben und Projektarbeit, wie z. B. die Planung und Durchführung von Patienten-Arzt-Seminaren, Symposien oder augenärztlichen Fortbildungen, die Übernahme von Verantwortung beim Qualitätsmanagement und die Einführung einer EPA, gehören zum „Homburger Curriculum“ [7].

Andere starke Aspekte von FIDUS sind nicht nur die sehr gute interne Organisation, sondern auch die gute Kommunikation zwischen den Ärzten an der Augenklinik und den niedergelassenen Augenärzten. FIDUSweb ermöglicht es, ein datenschutzkonformes Kooperationsportal mit einfachen Mitteln aufzusetzen. Durch die Umsetzung als Webportal lassen sich alle Endgeräte und elektronischen Patientenakten vernetzen. Besonders wichtige Funktionen sind die Arztbriefübermittlung in Echtzeit, Terminbuchungsfunktionalitäten, die Übermittlung von Daten der Bildgebung und die gemeinsame Behandlungsdokumentation [5]. Auch deshalb hat sich die Höchstbewertung hinsichtlich der „Transparenz der medizinischen Entscheidungen“ in unserer Klinik durch FIDUS von 2016 im Vergleich zu 2020 verdoppelt.

Unsere Studie belegt zweifelsfrei, dass die EPA FIDUS die Übersichtlichkeit der Akte im Vergleich zur Papierakte verbessert hat. Ein Mitarbeiter kann dem FIDUS-Team jederzeit einen Verbesserungsvorschlag mitteilen. Das FIDUS-Team bearbeitet den Vorschlag, und somit kann eine kontinuierliche Verbesserung von FIDUS erreicht werden. Die Arbeitsabläufe in unserer Klinik sind dank FIDUS schneller geworden. Die Nachvollziehbarkeit von Arbeitsabläufen und Anforderungen ist durch FIDUS besser geworden. Außerdem hat FIDUS die Transparenz der medizinischen Entscheidungen in unserer Klinik erhöht. Darüber hinaus hat FIDUS zu Zeitersparnissen im Arbeitsumfeld jedes Mitarbeiters geführt, und dadurch wurde die tägliche Arbeit vereinfacht sowie Überstunden der Assistenzärztinnen und -ärzte reduziert. Des Weiteren sind die Akteneinträge mit dem Computer schneller zu schreiben als handschriftlich. Klar im Vorteil ist hierbei zweifellos, wer eine 10-Finger-Schreibmaschinentechnik erlernt hat. Zusammenfassend wurde in der Studie deutlich, dass die Einführung der elektronischen Patientenakte der „richtige Schritt“ war. Ein solcher Schritt Richtung Digitalisierung ist für jede Klinik und Praxis empfehlenswert und unterstützungswürdig.

Darüber hinaus wurde die Arztbrieferstellung durch FIDUS ab 2016 als schneller bewertet im Vergleich zum vorherigen System. Die Anzahl der Höchstbewertung hat von 23,3 % auf 44,4 % deutlich zugenommen. Dies lässt sich dadurch erklären, dass die Briefvorlagen nach Einführung bedarfsgerecht angepasst und optimiert wurden. Da die Last der Papier-Altakten nach Einführung der EPA kontinuierlich abgenommen hat, ist die damit verbundene Belastung gesunken, was sich auch in der besseren Bewertung 2021 im Vergleich zu 2016 widerspiegelt.

Trotz der überwiegend positiven Resonanz zeigen sich auch einige Schwächen der elektronischen Patientenakte, die das FIDUS-Team als Anregung für weitere Optimierungen in den kommenden Monaten zur Kenntnis genommen hat. Einige Voraussetzungen sollen dafür in der nahen Zukunft durch das FIDUS-Team geschaffen werden. In unserer Studie gab es keinen signifikanten Unterschied bezüglich der Kommunikation in der Klinik zwischen 2016 und 2020. Dies kann dahingehend erklärt werden, dass die Kommunikation zwischen verschiedenen Mitarbeiterinnen und Mitarbeitern der Klinik vorwiegend „aktiv“ im persönlichen Gespräch und nicht durch FIDUS erfolgt. Außerdem gab es hinsichtlich der Abrechnung, des Einscannens der Tages- und Altakten innerhalb von 4 Jahren keinen signifikanten Unterschied. Ein Grund dafür ist, dass diese Prozeduren nicht nur auf FIDUS basieren, sondern auf andere parallel genutzte Softwaresysteme, wie z. B. SAP, zurückgreifen bzw. die Schnittstellenproblematik bei einem anderen Dienstleister liegt.

Nicht nur die ärztliche Dokumentation, sondern auch die Transparenz und Nachvollziehbarkeit von Anordnungen sowie der Befundaustausch mit niedergelassenen Kollegen ist durch FIDUS vereinfacht worden. Nach kurzer Eingewöhnungsphase stieß die Software rasch auf breite Akzeptanz. Auch im niedergelassenen Bereich wächst die Anzahl der FIDUS-Nutzer stetig. Insbesondere die digitale Terminvergabe und -planung, die Suchfunktion und die Möglichkeit für externe Partnerpraxen, Befunde einzusehen, fielen positiv ins Gewicht.

Die manuelle Übertragung von Daten aus Patientenmessgeräten in die elektronische Patientenakte (EPA) ist eine intensive, fehleranfällige Aufgabe, die das Pflegepersonal von der Patientenversorgung ablenkt und sich gleichzeitig negativ auf die Arbeitsleistung und die Mitarbeiterzufriedenheit auswirkt [4]. In der Publikation von Bauer et al. wurde Analytik zur Leistungsverbesserung verwendet, um angepasste Sätze von manuellen und automatisierten EPA-Dateneinträgen für 1933 aufeinanderfolgende Vitaldatenaufzeichnungen zu vergleichen, die von 49 Pflegekräften und zertifizierten Pflegeassistenten in einer 23-Betten-Einheit für medizinische Chirurgie in einem großen tertiären Krankenhaus erstellt wurden. Die Auswirkungen auf die Leistung und Qualität wurden anhand der Antworten des Pflegepersonals in einer Umfrage nach der Intervention bewertet. Sie kamen zu folgenden Ergebnissen: Die Datenfehler sind von ca. 20 % auf 0 gesunken; die Datenübertragungszeiten wurden um 5 min auf 2 h pro Messereignis reduziert; das Pflegepersonal hatte mehr Zeit für die direkte Patientenversorgung, und die Arbeitszufriedenheit verbesserte sich. Die Automatisierung der Dateneingabe eliminiert Datenfehler, reduziert die Verzögerungen bei der Dateneingabe in die EPAs erheblich und verbessert die Arbeitszufriedenheit, da das Pflegepersonal mehr Zeit für die direkte Patientenversorgung hat. Die Ergebnisse stehen im Zusammenhang mit Verbesserungen der Qualität, der Arbeitsleistung und der Arbeitszufriedenheit [7].

## Ausblick

In der Zukunft wird die Weiterentwicklung von FIDUS die Patientenversorgung weiter optimieren und telemedizinische Versorgungskonzepte ermöglichen. Die Grundlagen dazu sind unter anderem durch die gemeinsamen Verbesserungsvorschläge, die gute Kommunikation zwischen den Mitarbeiterinnen und Mitarbeitern und dem Softwarehersteller durch das FIDUS-Team sowie die Datenübermittlung über das Webportal „UKS.AUGEN.NETZ“ gelegt [5]. Dies steht im Einklang mit den Wünschen der Mitarbeiterinnen und Mitarbeiter. Mehr als die Hälfte davon sind der Meinung, dass die Ausweitung von FIDUS auf die Pflege sowie die digitale Patientenaufklärung wichtige nächste Schritte seien.

## Limitationen

Als Limitationen unserer Studie sind folgende Punkte zu erwähnen. Das Team im Jahr 2020 ist nicht mehr das gleiche wie 2016. Das Alter und der Berufsabschluss der Teilnehmer wurden nicht erhoben. Die Ergebnisse der Studie könnten dadurch beeinflusst worden sein, da z. B. ein älterer Teilnehmer mehr Vorbehalte gegenüber der elektronischen Lösung haben könnte als ein neu eingestellter, jüngerer Mitarbeiter, der keine Alternative kennen gelernt hat.

## Schlussfolgerung

- Die Zustimmungswerte zur elektronischen Patientenakte FIDUS haben sich 2020 im Vergleich zu 2016 signifikant verbessert.
- Das gilt insbesondere für die Bewertung der Übersichtlichkeit der EPA, hier hat sich die Anzahl der Höchstbewertungen (10) verdreifacht.
- Im Jahr 2020 hat sich die Mehrzahl der Befragten positiv zur Verkürzung der Bearbeitungsdauer bei Nutzung der computergestützten Akte im Vergleich zur handschriftlichen Ausarbeitung geäußert.
